# Ropinirole alters gene expression profiles in SH-SY5Y cells: a whole genome microarray study

**DOI:** 10.1590/1414-431X20154857

**Published:** 2016-01-19

**Authors:** M.Z. Zhu, W.D. Le, G. Jin

**Affiliations:** 1 School of Public Health, Shanghai University of Traditional Chinese Medicine, Shanghai, China; 2 Institute of Health Sciences, Shanghai Institutes for Biological Sciences, Chinese Academy of Science/Shanghai Jiao Tong University School of Medicine, Shanghai, China; 3 Department of Neurology, Baylor College of Medicine, Houston, TX., USA; 4 ShanghaiBio Corporation, North Brunswick, NJ, USA; 5 Shanghai Biochip Co., Ltd and National Engineering Center for Biochip at Shanghai, Shanghai, China

**Keywords:** Parkinson's disease, Gene expression, Ropinirole, SH-SY5Y

## Abstract

Ropinirole (ROP) is a dopamine agonist that has been used as therapy for Parkinson's disease. In the present study, we aimed to detect whether gene expression was modulated by ROP in SH-SY5Y cells. SH-SY5Y cell lines were treated with 10 µM ROP for 2 h, after which total RNA was extracted for whole genome analysis. Gene expression profiling revealed that 113 genes were differentially expressed after ROP treatment compared with control cells. Further pathway analysis revealed modulation of the phosphatidylinositol 3-kinase (PI3K) signaling pathway, with prominent upregulation of *PIK3C2B*. Moreover, batches of regulated genes, including *PIK3C2B*, were found to be located on chromosome 1. These findings were validated by quantitative RT-PCR and Western blot analysis. Our study, therefore, revealed that ROP altered gene expression in SH-SY5Y cells, and future investigation of *PIK3C2B* and other loci on chromosome 1 may provide long-term implications for identifying novel target genes of Parkinson's disease.

## Introduction

Parkinson's disease (PD) is a progressive neurological disorder with primary symptoms of bradykinesia, tremor, and rigidity, and patients with advanced disease also show postural instability. It is the second most common neurodegenerative disorder after Alzheimer's disease, and is responsible for significant morbidity as well as shortened life expectancy. It also places a substantial economic burden on the patient, their family, and the society (1). Therefore, any therapy proven to modify the course of PD would be extremely valuable.

To date, treatments for PD have been confined to symptomatic therapies, which have focused on motor deficits and the loss of dopaminergic neurons in the substantia nigra. However, the past few years have seen important advances in the development of new drugs for PD, and importantly how existing drugs are used as part of a long-term strategy for disease management (2).

Ropinirole (ROP) is a novel dopamine receptor agonist with a high affinity for all dopamine D2 subfamily receptors, but the highest affinity for the D3 receptor subtype (3). It has been demonstrated to have neuroprotective effects and has been used for clinical PD therapy (4). Researchers have made many attempts to clarify the potential mechanism of ROP action over recent years. For instance, it has been thought to increase the concentration of glutathione, catalase, and superoxide dismutase (5). Moreover, both bromocriptine and ROP were shown to reduce hydroxyl radical generation in the rodent striatum after infusion of the neurotoxin 1-methyl-4-phenylpyridinium (6). However, the exact mechanisms appear complicated and controversial and require further investigation. The advent of microarray chips provides an entirely new approach to the molecular characterization of neurodegenerative diseases and their models (7).

In the present study, therefore, we used genome-wide Affymetrix microarrays to identify genes that were regulated following ROP treatment of SH-SY5Y cells to illustrate the potential mechanisms of its effects.

## Material and Methods

### Cell culture

The human neuroblastoma cell line SH-SY5Y, subcloned from the SK-N-SH cell line, is often used as a model of human dopaminergic neurons, and thus was used in the current study. Cells were routinely cultured in Dulbecco's modified Eagle's medium supplemented with 10% heat-inactivated fetal bovine serum (FBS, Gibco, USA) and maintained at 37°C under a humidified 5% CO_2_ atmosphere. ROP stock was freshly made in water prior to each experiment, and cells were divided into two groups (control and treated groups). Cells of treated groups were exposed for 2 h to 10 µM ROP, which is thought to be a clinically relevant dose (8). Control cells received no ROP treatment.

### Total RNA extraction and microarray experiments

Total RNA fractions were isolated from cultured cells after specific treatment using the SV total RNA isolation system (Promega, USA). Total RNA samples were spectrophotometrically scanned from 220 to 320 nm; A260/A280 was typically >1.9. Formaldehyde agarose gel electrophoresis was used as a quality control for total RNA. RNA samples were then used to generate biotinylated cRNA targets for the Affymetrix GeneChip Human genome U133 Set. Six microarray chips were prepared, including three biological replicates for control and treated groups. All experiments were performed according to manufacturer protocols (Affymetrix Inc., USA).

After hybridization, arrays were stained in the GeneChip Fluidics Station 450 and scanned on the Affymetrix Scanner 3000. Fluorescent signal intensities were analyzed using the Gene Chip Operating System (Affymetrix). Ratios comparing treated and control groups were calculated to represent fold-changes in gene expression. Regulated genes were shown to be consistent across all biological replicate sets. Annotation and categorization of the regulated genes were based on gene ontology performed by the Database for Annotation, Visualization and Integrated Discovery (DAVID; https://david.ncifcrf.gov/summary.jsp). KEGG and BIOCARTA functional pathways were evaluated for regulated genes, and DAVID 2007 was used to detect chromosomal loci containing ROP-modulated genes.

### TaqMan^¯^ real-time PCR assay

Total RNA was isolated as described above and reverse transcription was performed using the High Capacity cDNA Reverse Transcription kit (Applied Biosystems, USA). PCR amplification was performed using TaqMan Gene Expression Assays and the TaqMan Universal PCR Master Mix according to the manufacturer's instructions (Applied Biosystems). Assay IDs of genes *CALM3*, *EPS15*, *RIPK5*, and *PIK3C2B* were Hs00270914_m1, Hs00179978_m1, Hs00418647_m1, and Hs00153248_m1, respectively. Amplification was conducted on duplicate samples using the ABI 7900 Detection System according to the manufacturer's instructions. *GAPDH* was used as an endogenous control to normalize all assays. Relative quantification of gene expression levels was determined using the Comparative Ct method.

### Western blot analysis

Proteins were extracted using RIPA Lysis Buffer (Beyotime Institute of Biotechnology, China) with Phosphatase Inhibitor Cocktail Tablets (Roche, Switzerland) according to the manufacturer's instructions. Briefly, 100 µg of protein was run on a 12% denaturing polyacrylamide gel and transferred onto a polyvinylidene fluoride membrane. After incubation with an anti-PIK3C2B primary antibody (Abcam, USA), the membrane was washed and incubated with a corresponding horseradish peroxidase-labeled secondary antibody (BD Pharmingen, USA). Detection was performed using an ECL kit (Amersham Pharmacia Biotech, Japan) according to the manufacturer's instructions. Absorbance was analyzed using Image-Pro Plus software (Media Cybernetics, USA). PIK3C2B protein levels were was normalized to those of β-actin and compared among groups.

### Statistical analysis

Data are reported as means±SE. Statistical analysis was performed using analysis of variance and the Student's *t*-test. Genes were deemed significantly different between groups if P<0.05, and if fold-changes were greater than 1.5 or less than 0.67. P values were corrected by the Benjamini-Hochberg false discovery rate method using R software.

## Results

### ROP modulated gene expression in SH-SY5Y cells

**Figure 1:**
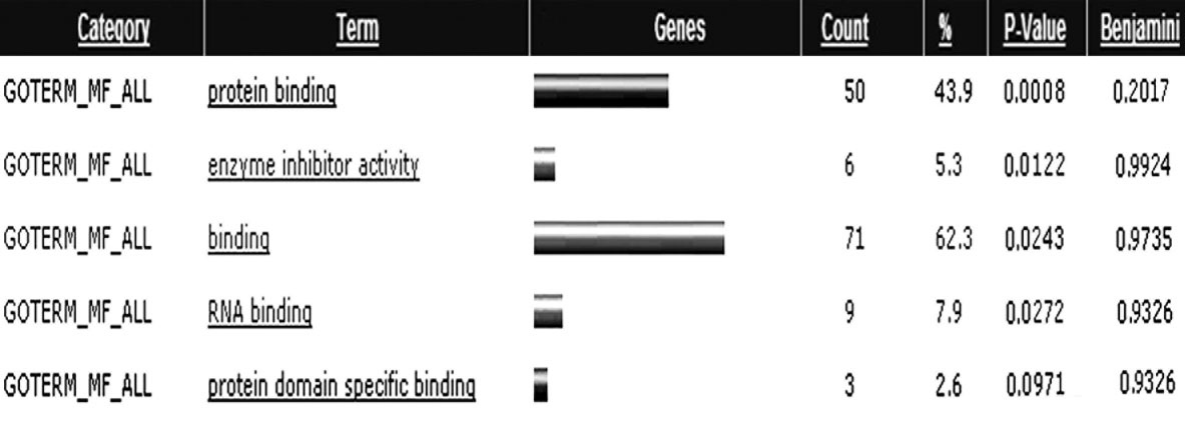
Molecular function categories of genes regulated by ropinirole (ROP). GOTERM_Molecular Function_ALL revealed that most of these genes functioned in protein and RNA binding, and in enzyme inhibitor activity.

We identified a total of 113 genes as differentially regulated by ROP treatment, of which 48 were upregulated and 65 were downregulated. Among the 113 genes, 101 were known genes and 12 were expressed sequence tags. As shown in Figure 1, GOTERM_Molecular Function_ALL revealed that most of these genes had functions in protein and RNA binding, and enzyme inhibitor activity. Table 1 lists 20 genes representative of the complete list. Further pathway analysis (KEGG and BIOCARTA functional pathways) revealed that only the phosphatidylinositol 3-kinase (PI3K) signaling pathway was over-represented, including genes *CALM3*, *INPP4A*, and *PIK3C2B*. Notably, *PIK3C2B* expression was strongly promoted by ROP treatment in this pathway. We also identified a number of modulated genes that are located near *PIK3C2B* on chromosome 1, including *KLHL17*, *USP24*, *C1ORF149*, *ID3*, *MTHFR*, *KIAA0090*, *ADAMTSL4*, *SERBP1*, *RIPK5*, *EPS15*, *PIGR*, *ZFYVE9*, and *ZMYM6*. Of these, *EPS15* expression was clearly induced by ROP (Table 2).

**Table:**
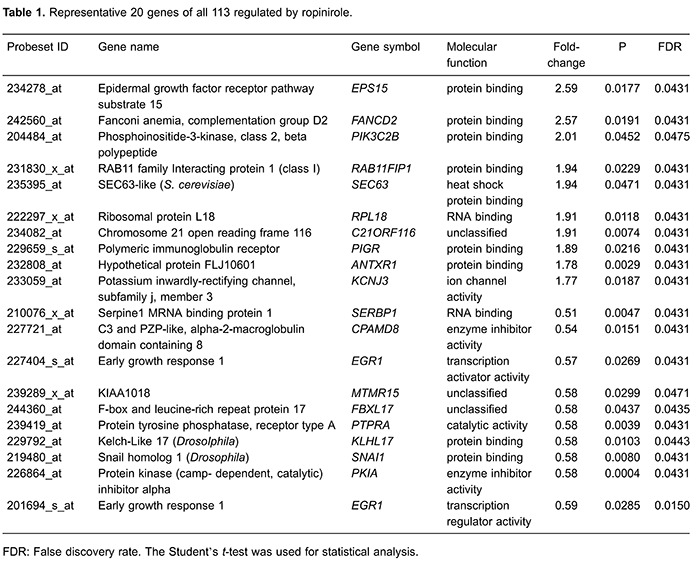

**Table:**
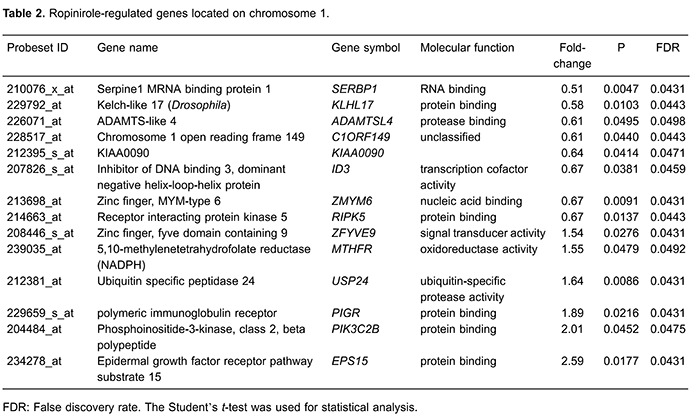

### TaqMan real-time PCR and Western blot

**Figure 2:**
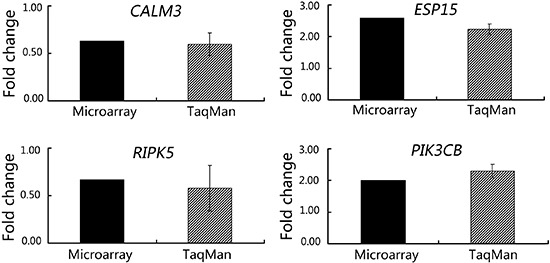
TaqMan¯ real-time PCR confirmation of microarray data in SH-SY5Y cells. *CALM3*, *EPS15*, *RIPK5*, and *PIK3C2B* underwent TaqMan real-time PCR in SH-SY5Y cells. The y-axis represents the fold-change in expression after ROP treatment, and microarray and TaqMan data are plotted on the x-axis. There were no significant differences between the microarray and TaqMan data (P>0.05; Student's *t-*test).

**Figure 3:**
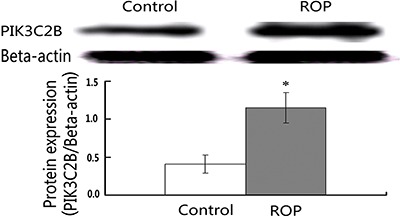
Elevation of PIK3C2B protein expression by ropinirole (ROP) treatment in SH-SY5Y cells. β-actin was used as the loading control. PIC3C2B protein levels were significantly increased by treatment with 10 µM ROP. *P<0.05, 10 µM ROP compared to the control group (Student's *t*-test).

To confirm this observed regulation of gene expression by ROP treatment, we performed TaqMan real-time PCR of a number of selected genes. *CALM3* was chosen because it is involved in the PI3K signaling pathway; *PIK3C2B* was selected for its prominent elevation and potential role in PD; and *EPS15* and *RIPK5* were selected as representative loci on chromosome 1 and their putative relationship with PD. As shown in Figure 2, a strong increase in the mRNA levels of *PIK3C2B* and *EPS15* was detected following ROP treatment, which supported microarray data. A change in expression of *CALM3* and *RIPK5* was also confirmed. Western blotting showed that PIK3C2B protein levels were 2.5 times higher in the ROP-treated group than the control group (Figure 3).

**Figure 4:**
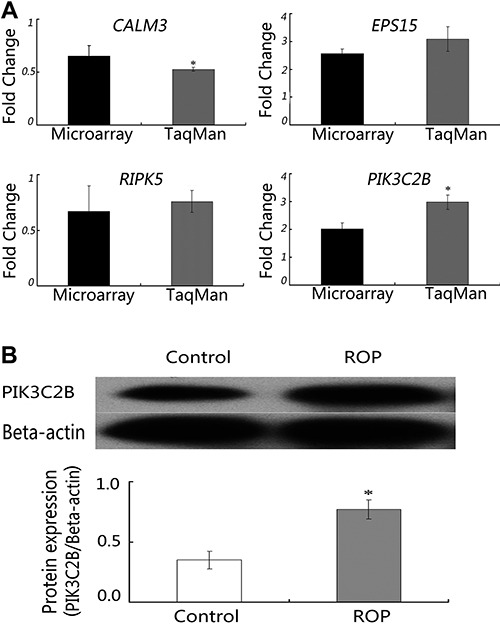
Validation of microarray data in HeLa cells. *A*, *CALM3*, *EPS15*, *RIPK5*, and *PIK3C2B* underwent TaqMan*¯* real-time PCR in HeLa cells. The y-axis represents the fold-change in expression after ROP treatment, and microarray and TaqMan data are plotted on the x-axis. *P<0.05, TaqMan data compared to microarray data (Student's *t*-test). *B*, Western blot analysis in HeLa cells. β-actin was used as the loading control. PIC3C2B protein levels were elevated by treatment with 10 µM. *P<0.05, 10 µM ROP compared to control (Student's *t*-test).

We validated our microarray data in HeLa cells by performing cell culture, TaqMan real-time PCR, and Western blotting as described previously. As shown in Figure 4, similar expression patterns were observed in HeLa cells to those seen in SH-SY5Y cells.

## Discussion

PD is a neuropathological disorder involving the degeneration of dopaminergic neurons in the substantia nigra, and subsequent loss of their terminals in the striatum. The ensuing loss of dopamine causes most of the debilitating motor disturbances associated with PD. Current PD medications treat the symptoms of the disease, focusing on halting or retarding the degeneration of dopaminergic neurons. Recently, there has been considerable interest in neuroprotection as a therapeutic strategy for PD, and several drugs such as ROP have been proposed as candidate agents (9). However, the molecular mechanism of neuroprotection is elusive.

In the present study, we treated SH-SY5Y cells with ROP and applied whole-genome microarray to screen changes in gene expression with the aim of uncovering the underlying molecular mechanism. Using bioinformatics, we identified genes that were differentially regulated after ROP treatment, which are known to function in protein and RNA binding, and enzyme inhibitor activity. We also observed that the PI3K signaling pathway was over-represented and that *PIK3C2B* expression was distinctly increased in this pathway.

The PI3K family is evolutionarily conserved and is implicated in many biological processes including cell survival, proliferation, inflammation, adhesion, glucose metabolism, chemotaxis, and cancer. It can be classified into three distinct sub-groups (I, II, and III) based on substrate specificity and sequence homology. PIK3C2B is a family member of class II proteins, which contain a C2 domain and PX domain (10,11). Although diverse biological roles have been assigned to class I and class III PI3Ks, the functions of class II PI3Ks are still unknown. However, PIK3C2B has recently been implicated in cell growth, cell migration, and differentiation (12 13 14). Moreover, activation of a major neuroprotective signaling pathway, the PI3K/Akt pathway, can prevent cell death in a PD model of SH-SY5Y cells (9).

Intriguingly, we observed the distinct promotion of *PIK3C2B* transcript and protein expression levels in SH-SY5Y cells following ROP treatment. This indicated that ROP might exert neuroprotective effects through the PI3K pathway, and that PIK3C2B might play a role in this process. Additionally, we previously observed that the PI3K/Akt pathway modulates the expression of Nurr1, which is a transcription factor essential for the differentiation and maturation of central dopaminergic cells (15). This suggested that ROP might induce PIK3C2B and modulate Nurr1 to exert neuroprotection. However, the present study found no direct evidence of Nurr1 modulation by either ROP or PIK3C2B. Further investigations may shed new light on the mechanism of ROP neuroprotection and the role of PIK3C2B in PD.

Nine loci in the human genome have previously been linked to PD. Mutations in alfa-synuclein, parkin, *DJ-1*, and, arguably *UCH-L1* genes have been associated with familial PD (16). Recently a locus on chromosome 1 was linked to common late-onset PD in the Icelandic population (16). Meanwhile, linkage studies have also defined susceptibility regions for late-onset PD on chromosomes 1 and 2 (17). We observed that ROP regulated several genes located on chromosome 1, suggesting that this might be its main way of exerting neuroprotective effects. Only three of these genes, *USP24*, *MTHFR*, and *EPS15*, have been associated with PD in earlier studies (17 18 19). For instance, *in vitro* experiments showed that *EPS15* enhanced the ubiquitin ligase activity of PARKIN, and PARKIN-mediated EPS15 ubiquitination is crucial in promoting the PI3K/Akt signaling pathway (19,20).

In the present study, we observed that ROP distinctly increased the expression of *EPS15*. Considering the role of EPS15 and PI3K/Akt in neuronal survival, our observation is likely to further our understanding of the role of ROP in PD therapy. Despite other chromosomal 1 genes having no known link with PD, their underlying biological functions may nevertheless provide new implications for disease. RIPK5, a member of the RIP serine/threonine kinase family, was previously reported to induce both caspase-dependent apoptosis and caspase-independent cell death (21). Considering the important role of cell death pathways in PD (22), future work may identify a novel role for RIPK5 in the pathogenesis of PD.

In conclusion, we used genome-wide microarray analysis to identify genes that were regulated after ROP treatment. Pathway analysis suggested that ROP mainly modulated the PI3K signaling pathway in SH-SY5Y cells. Further extensive investigation of *PIK3C2B* and other loci on chromosome 1 may open up a new avenue to understand the pathology of PD and provide novel pharmaceutical targets to improve patient care.
